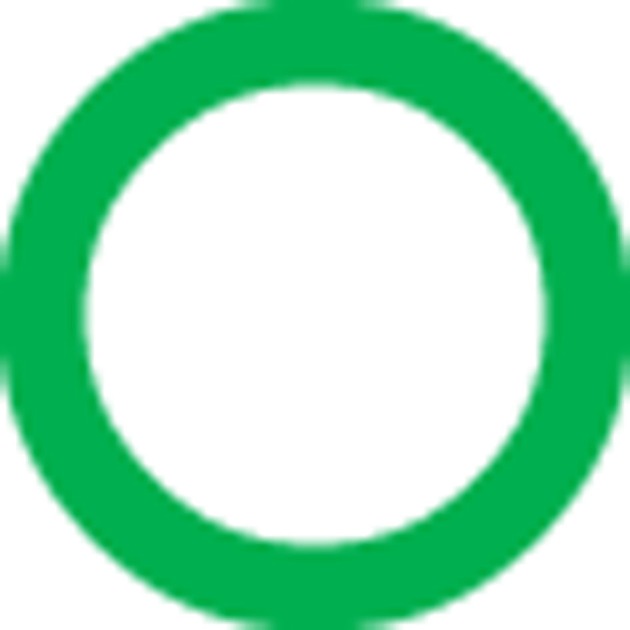# Layer-specific cholinergic control of human and mouse cortical synaptic plasticity

**DOI:** 10.1038/ncomms12826

**Published:** 2016-09-08

**Authors:** Matthijs B. Verhoog, Joshua Obermayer, Christian A. Kortleven, René Wilbers, Jordi Wester, Johannes C. Baayen, Christiaan P. J. De Kock, Rhiannon M. Meredith, Huibert D. Mansvelder

**Affiliations:** 1 Department of Integrative Neurophysiology, Center for Neurogenomics and Cognitive Research, VU University Amsterdam, De Boelelaan 1085, Amsterdam 1081 HV, The Netherlands; 2 Department of Neurosurgery, Neuroscience Campus Amsterdam, VU University Medical Center Amsterdam, De Boelelaan 1117, Amsterdam 1081 HV, The Netherlands

## Abstract

Individual cortical layers have distinct roles in information processing. All layers receive cholinergic inputs from the basal forebrain (BF), which is crucial for cognition. Acetylcholinergic receptors are differentially distributed across cortical layers, and recent evidence suggests that different populations of BF cholinergic neurons may target specific prefrontal cortical (PFC) layers, raising the question of whether cholinergic control of the PFC is layer dependent. Here we address this issue and reveal dendritic mechanisms by which endogenous cholinergic modulation of synaptic plasticity is opposite in superficial and deep layers of both mouse and human neocortex. Our results show that in different cortical layers, spike timing-dependent plasticity is oppositely regulated by the activation of nicotinic acetylcholine receptors (nAChRs) either located on dendrites of principal neurons or on GABAergic interneurons. Thus, layer-specific nAChR expression allows functional layer-specific control of cortical processing and plasticity by the BF cholinergic system, which is evolutionarily conserved from mice to humans.

## Abstract (web summary)

Nicotinic acetylcholine receptors (nAChRs) are differentially expressed across cortical layers, yet it is unclear whether they show layer-specific effects on synaptic plasticity in the prefrontal cortex. Here, the authors compare nAChRs across L6 and L2/3 in human and mouse cortex and find they mediate opposite effects on synaptic plasticity.

## Graphical abstract

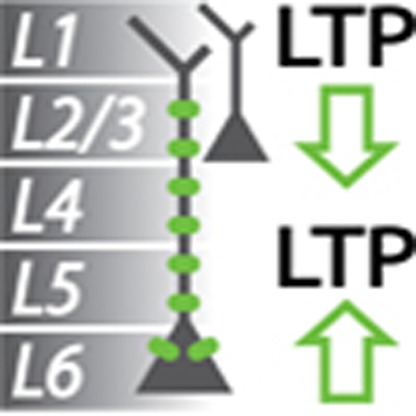

Cortical acetylcholine (ACh) signalling shapes neuronal circuit development and underlies specific aspects of cognitive functions and behaviors, including attention, learning, memory and motivation^1^,^2^,^3^,^4^,^5^,^6^. On the basis of anatomical findings, control of cortical processing by projections from sparse cholinergic nuclei in the basal forebrain (BF) could be much more specific than classically thought^7^,^8^. Within the mouse BF, a topographic organization exists by which different areas of the medial prefrontal cortex (mPFC) are innervated by different BF cholinergic neurons^7^. Moreover, these neurons preferentially target superficial or deep cortical layers^7^. Both muscarinic and nicotinic ACh receptors (mAChRs and nAChRs) are expressed in a layer-dependent fashion as well^9^,^10^,^11^, opening the possibility that cholinergic control of cortical processing is layer specific. Indeed, the distinct, layer-dependent expression of nAChRs in the mPFC could support a layer-dependent control of excitability of pyramidal neurons by cholinergic projections from the BF^11^,^12^. Applications of ACh show that superficial layer 2/3 (L2/3) pyramidal neurons are inhibited by nAChR activation on interneurons, while deep L6 pyramidal neurons are excited by postsynaptic nAChRs^11^,^13^,^14^,^15^.

The cellular and sub-cellular location of nAChRs may not only determine how excitability in neuronal circuitries is affected, but may also decide how plasticity of glutamatergic synapses is affected by cholinergic inputs. Activation of nAChRs located on presynaptic terminals can increase glutamate release from synapses^16^,^17^. In particular, presynaptic nAChRs containing α7 subunits, which have high calcium permeability^18^, can cause long-term potentiation of glutamatergic synapse strength in different brain regions^19^,^20^,^21^. In the PFC, despite the expression of α7-containing nAChRs on L5 pyramidal neurons and strong transient modulation of thalamic excitatory inputs by nAChR activation^11^,^22^,^23^, nAChRs located on GABAergic interneurons augment inhibitory synaptic transmission and reduce excitability of L5 pyramidal neuron dendrites, thereby suppressing long-term potentiation of glutamatergic synapses^23^,^24^. In contrast to L5, inhibitory GABAergic and excitatory glutamatergic transmission onto PFC L6 pyramidal neurons are not modulated by nAChRs^11^. Instead, L6 pyramidal neurons express nAChRs themselves and are therefore directly activated by ACh^11^,^13^,^14^. These findings suggest that the mechanisms by which nAChRs alter synaptic plasticity of glutamatergic synapses in L5 pyramidal neurons may not be in place in L6.

It is not known whether postsynaptically located nAChRs in the PFC modulate long-term plasticity of glutamatergic synapses and whether this can be induced by endogenous ACh release. Here we find that endogenous ACh release modulates cortical plasticity rules with layer specificity: in L6 endogenous ACh release modulates plasticity in the opposite direction from superficial layers. Endogenous ACh augments long-term strengthening of glutamatergic synapses on L6 pyramidal neurons by activating heteromeric postsynaptic nAChRs containing β2 and α5 subunits. In addition, we find these mechanisms also operate in the human neocortex, where layer-specific expression of functional nAChRs supports opposite cholinergic modulation of synaptic plasticity in superficial and deep cortical layers.

## Results

### Nicotine facilitates tLTP of L6 pyramidal neuron synapses

**Figure 1:**
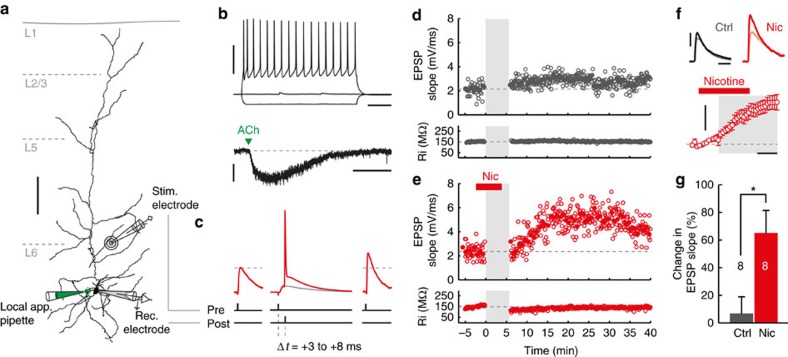
Nicotine facilitates tLTP in L6 pyramidal neurons. (**a**) Biocytin reconstruction of layer 6 pyramidal neuron from coronal slice of mouse prelimbic mPFC showing relative positions of recording and stimulating electrodes, and local application pipette. Scale bar, 100 μm. (**b**) Voltage responses to hyperpolarizing (−120 pA) and depolarizing (+330 pA) somatic current injections (top traces) and current response to local application of acetylcholine (1 mM, 10 ms, bottom trace) to soma of neuron in **a**. Top scale bars, 40 mV, 100 ms; bottom scale bars, 50 pA, 1 s. (**c**) Plasticity induction protocol. EPSPs were evoked by extracellular stimulation 100–150 μm from soma along the apical dendrite (**a**). After obtaining a baseline measure of EPSP (left trace), tLTP was induced by repeatedly pairing EPSPs to APs (middle traces; +3 to 8 ms delay, 50 repetitions). EPSPs were then recorded for up to 30 min. to observe changes in EPSP slope (right trace). (**d**) Example of a tLTP experiment in control conditions showing slope and input resistance (top and bottom panels, respectively) versus time. Grey shading indicates time of EPSP+AP pairing. Open grey circle=single EPSP, grey circle=mean of 7 EPSPs. (**e**) As **d**, for a tLTP experiment where nicotine (300 nM) was bath-applied during pairing (red bar). 
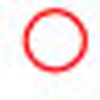

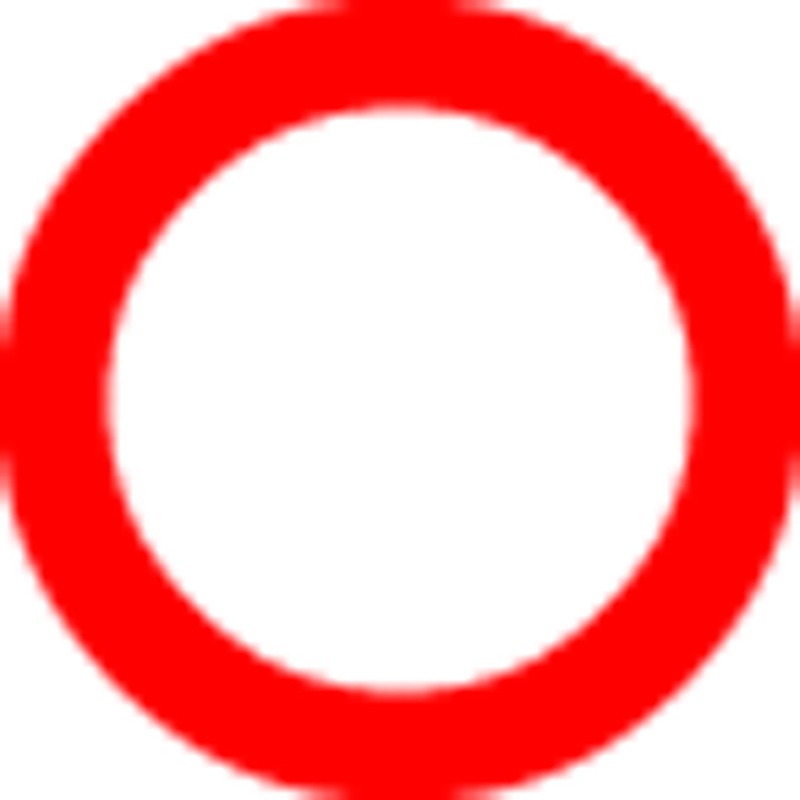
open red circle=single EPSP, red circle=mean of 7 EPSPs. (**f**) Top: Example EPSP waveforms recorded during baseline (light color) and 20–25 min. after pairing (dark color), for control and nicotine tLTP experiments shown in (**d**,**e**), respectively (scale bars, 3 mV, 30 ms). Bottom: membrane potential change relative to baseline over the course of pairing (grey shading) for STDP experiments where nicotine was applied during pairing (*n*=8 cells (five animals), mean±s.e.m. in 14 s bins). Scale bars, 5 mV, 2 min. (**g**) Summary bar chart of control and nicotine tLTP experiments, showing percentage change in EPSP slope for both conditions (mean±s.e.m.; control: *n*=8 (seven animals), nicotine: *n*=8 (five animals) one-way ANOVA: F_(1,15)_=7.242, *P*=0.017).

Nicotinic ACh receptors located at presynaptic locations can alter synaptic plasticity of glutamatergic synapses^19^,^23^,^24^. To test whether postsynaptic nAChRs affect synaptic plasticity of glutamatergic synapses, whole-cell recordings were made from L6 pyramidal neurons of mouse prelimbic (PrL)-mPFC (P21–30) that express nAChRs (Fig. 1a,b). Layer 6 was identified under oblique illumination as a layer containing relatively densely packed neurons with small somata at a perpendicular distance of >∼650 μm from the pia. These neurons generally had regular spiking properties (Fig. 1b) and displayed strong inward currents in response to a local application of ACh (1 mM, 10 ms, in presence of 400 nM atropine, local application protocol I, Methods section) aimed at the cell body (Fig. 1b), as was shown previously^11^,^12^,^13^,^14^.

To investigate the effect of postsynaptic nAChR stimulation on long-term plasticity of glutamatergic synapses, excitatory postsynaptic potentials (EPSPs) were evoked every 7 s by extracellular stimulation 100–150 μm from soma along the apical dendrite (Fig. 1a,c). After obtaining a stable baseline measure of the EPSP waveform, EPSPs were repeatedly paired to postsynaptic action potentials (APs) evoked by brief somatic current injection with a delay of 3–8 ms (Fig. 1c; see Methods section for more details on the spike-timing-dependent long-term potentiation (tLTP) induction protocol). After pairing, EPSPs were recorded for up to 30 min to measure changes in EPSP slope. This protocol elicits robust tLTP in L5 pyramidal neurons of mouse PrL-mPFC^23^. In L6 pyramidal neurons however, only a mild potentiation of EPSPs was induced (Fig. 1d,f,g; ΔEPSP slope: +7±13%, *n*=8). To activate nAChRs, nicotine (300 nM) was washed into the bath 2 min before and during the first 3 min of plasticity induction, which resulted in a clear postsynaptic depolarization (4.1±1.0 mV, *n*=9, Fig. 1f bottom panel). Following these conditions, a strong lasting increase in EPSP slope was observed in response to pairing (Δslope: +65±17%, *n*=9), which was significantly larger than that observed in control conditions (Fig. 1e–g; *P*=0.017). Omission of the postsynaptic AP during pairing in presence of nicotine did not result in a significant change in EPSP slope (Δslope: +11±16%, *n*=4; paired samples *t*-test, *P*=0.791). These results show that in contrast to L5, acute exposure to nicotine at smoking-relevant concentrations facilitates tLTP of layer 6 pyramidal neuron synapses.

### Endogenous ACh modulates STDP with layer specificity

**Figure 2:**
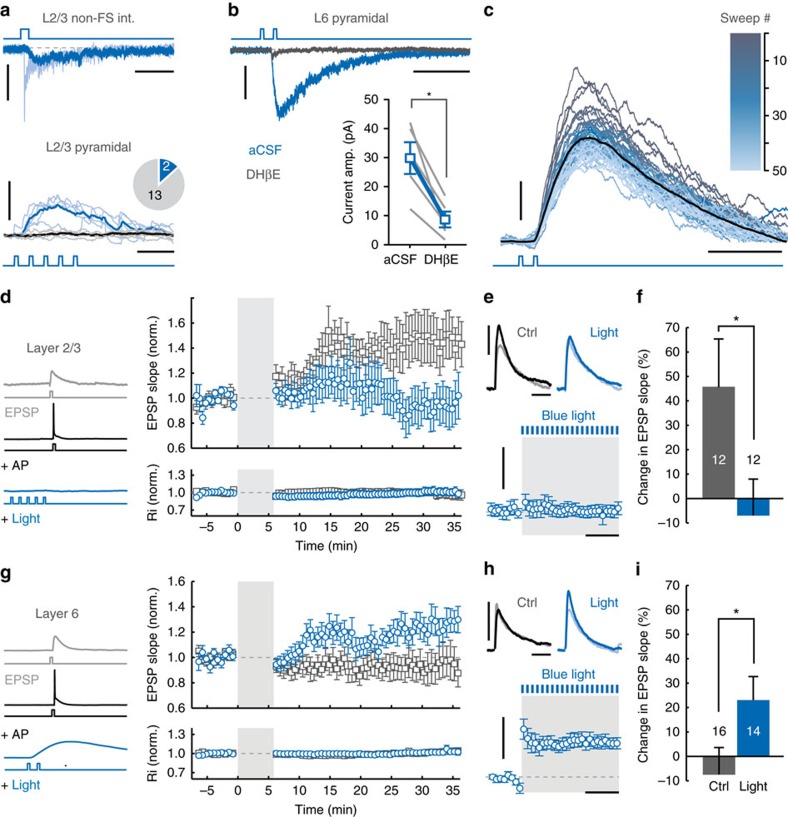
Endogenous Ach oppositely regulates STDP in superficial and deep neocortical layers. (**a**) Top: nAChR currents in L2/3 non-FS interneurons following activation of cholinergic fibers with blue light (470 nm, 100 ms). Scale bars, 20 pA, 500 ms. Bottom: Same, but in L2/3 pyramidal neurons (470 nm, 10 ms, 25 Hz). 2 of 15 neurons showed light-evoked potentials (blue traces; light: single trial, dark: average of 5 trials). Scale bars, 3 mV, 100 ms. (**b**) Light-evoked nAChR-mediated currents in L6 pyramidal neuron (470 nm, 10 ms, 25 Hz) in aCSF (blue trace), or with DHβE (10 μM, grey trace) in *Chat-ChR2(N6)* and *Chat-Cre/Ai32* mice. Right: summary of amplitudes in aCSF and with DHβE (*n*=5 (two animals), paired *t*-test, *P*=0.009). Scale bars, 20 pA, 200 ms. (**c**) Light-evoked nAChR-EPSPs in *Chat-ChR2(N6)* L6 pyramidal neurons (10 ms, 25 Hz), 50 times at 0.14 Hz. Mean nAChR-EPSP in black. Scale bars, 3 mV, 200 ms. (**d**) tLTP induction in L2/3 pyramidal neurons. Left: schematics of pairing and relative timing. Right: Summary of EPSP slope and input resistance for tLTP experiments (control open grey square, light-evoked ACh release open blue circle, mean±s.e.m. of 30 s bins). (**e**) Top: EPSP waveforms during baseline (light) and 20–25 min after pairing (dark), with and without light-evoked ACh release in L2/3 pyramidal neurons. Scale bars, 3 mV, 30 ms. Bottom: Vm at onset of EPSP during pairing period (grey shading), relative to baseline (*n*=12 (seven animals), mean±s.e.m., 14 s bins). Scale bars, 5 mV, 2 min. (**f**) Summary of control tLTP and with light-evoked endogenous ACh release in L2/3 pyramidal neurons (mean±s.e.m.). EPSP+AP pairing with light (*n*=12 (seven animals)): −7.0±14.9%; EPSP+AP pairing without light (*n*=12 (eight animals)): +45.8±19.6%. Independent samples *t*-test: *P*=0.044. (**g**) As **d**, for L6 pyramidal neurons. (**h**) As **e**, for L6 pyramidal neurons. (**i**) As **f**, for L6 pyramidal neurons. EPSP+AP pairing with light (*n*=14 (twelve animals)): Median=+27%, IQR=43%; EPSP+AP pairing without light (*n*= 16 (ten animals)): Median=−20%, IQR=60%. Independent samples Mann-Whitney *U*-test: *U*_(30)_=165, *P*=0.028.

Cortical nAChRs are activated by endogenous cholinergic projections from the BF^25^,^26^,^27^. To specifically activate cholinergic projections we used transgenic mice that expressed channel rhodopsin (ChR2) in Chat-positive neurons^28^, which allows for light-evoked ACh release from cholinergic terminals. We have previously shown that in L2/3, nAChRs are expressed predominantly by interneurons and only by a small fraction of pyramidal neurons^11^. Consistent with these reports, we observed nAChR-mediated currents in response to light in all L2/3 non-FS interneurons (10 out of 10) and in only a few L2/3 pyramidal neurons (2 out of 15; Fig. 2a). Conversely, in L6, light-evoked responses were observed in all pyramidal neurons. These currents were blocked by DHβE (Fig. 2b; aCSF: 30.0±5.5 pA versus DHβE: 8.8±2.7 pA, *n*=5, paired *t*-test, *P*=0.009), consistent with earlier reports that cholinergic fibers activate postsynaptic β2-containing nAChRs in L6 pyramidal neurons^29^,^30^.

To test whether the layer- and cell type-specific modulation of neuronal excitability by endogenous ACh results in layer-specific modulation of mPFC spike timing-dependent plasticity (STDP) rules, we performed tLTP experiments in L2/3 and L6 with light-evoked ACh release during plasticity induction. BF cholinergic neurons fire in short bursts *in vivo* during wakefulness^31^,^32^. To mimic naturalistic firing patterns of these neurons, blue light pulses of 10 ms each were delivered at 25 Hz (Fig. 2c), which is within the range of natural firing frequencies of BF cholinergic neurons observed *in vivo*^31^. In L2/3, EPSP+AP pairing without light resulted in a robust tLTP (+46±20%, *n*=12). However, endogenous ACh, released with a burst of blue light pulses preceding EPSP+AP pairing, prevented tLTP (−7±15%, *n*=12; Independent samples *t*-test: *P*=0.044; Fig. 2d–f).

In L6, activation of cholinergic fibers evoked robust nAChR-mediated EPSPs (nAChR–EPSPs) with limited rundown or desensitization over the course of 50 trials with a 7 s trial interval (Fig. 2c). To investigate whether endogenous ACh could modulate tLTP, light-induced ACh release was evoked shortly before each EPSP+AP pairing trial during plasticity induction, which caused the EPSP+AP to approximately coincide with the peak of the nAChR–EPSP (Fig. 2g). EPSP+AP pairing with coincident light-evoked ACh release resulted in tLTP (+23±10%, *n*=14), which was not seen in the control group of L6 pyramidal neurons from Chat-ChR2 mice that was not light-stimulated during pairing (Fig. 2g–i; −7±11%, *n*=16; Mann–Whitney *U*-test, *P*=0.028). Altogether, these results show that brief cholinergic signals on naturalistic timescales^31^ are sufficient to prevent tLTP of glutamatergic synapses in L2/3 pyramidal neurons, and to facilitate tLTP in L6 pyramidal neurons. Thus, endogenous cholinergic inputs modulate mPFC plasticity rules with layer specificity, having opposite effects in superficial versus deep cortical layers.

### Facilitation of tLTP in L6 requires α5 nAChR subunits

**Figure 3:**
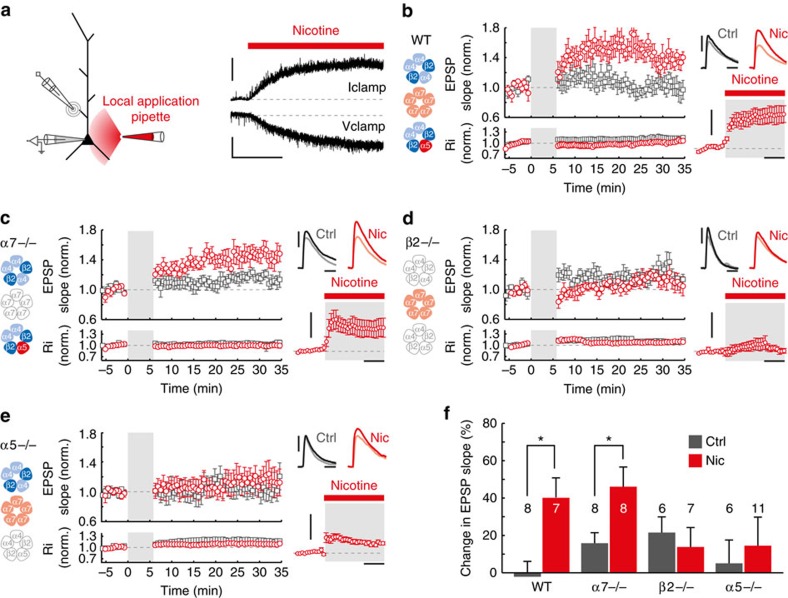
Facilitation of tLTP in L6 depends on nAChRs with β2 and α5 subunits. (**a**) Recording configuration for plasticity experiments. nicotine (10 μM) was delivered by local application at somato-dendritic regions. Right traces: voltage (top) and current (bottom) responses to local application in WT L6 pyramidal neuron. Scale bars, 4 mV (top), 20 pA (bottom), 30 s. (**b**) Summary of nicotine application during EPSP-AP pairing in WT L6 pyramidal neurons (One-way ANOVA: F_(1,13)_=10.129, *P*=0.007; control: *n*=8 (seven animals), nicotine: *n*=7 (five animals)). Left: Predominant types of nAChRs in cerebral cortex; α4β2*-nAChRs (top), α7-nAChRs (middle) and α4β2α5*-nAChRs (bottom). Middle panels: EPSP slope (top) and input resistance (bottom) of control (open grey square circle) and nicotine (open red circle) tLTP experiments, normalized to baseline (mean±s.e.m., 30 s bins). Right traces, top: EPSP waveforms recorded during baseline (light color) and 20–25 min. after pairing (dark color), control and nicotine tLTP experiments (scale bars, 3 mV, 30 ms). Right traces, bottom: Vm change relative to baseline by local nicotine application (grey shading) for tLTP experiments (mean±s.e.m., 14 s bins). Scale bars, 5 mV, 2 min. (**c**) As (**b**), for mice lacking α7-nAChRs (α7*−/−*). One-way ANOVA: F_(1,14)_=6.418, *P*=0.024; control: *n*=8 (four animals), nicotine: *n*=8 (six animals). (**d**) As (**b**), for mice lacking β2 subunit-containing nAChRs (β2−/−). One-way ANOVA: F_(1,11)_=0.316, *P*=0.585; control: *n*=6 (five animals), nicotine: *n*=7 (four animals). (**e**) As (**b**), for mice lacking α5 subunit-containing nAChRs (α5−/−). control: Median=13%, IQR=60%; nicotine: Median=+2%, IQR=40%; Mann–Whitney *U*-test: *U*_(17)_=31, *P*=0.884; control: *n*=6 (five animals), nicotine: *n*=11 (six animals). (**f**) Summary of control tLTP and nicotine tLTP experiments, showing change in EPSP slope for experiments in WT animals and the three nAChR knockout mouse lines tested (mean±s.e.m.).

Previous work has shown that L6 pyramidal neurons express nAChRs that contain β2 and α5 subunits^11^,^13^,^15^. However, a small fraction (20%) of L6 pyramidal neurons additionally express α7 nAChRs^11^. To test which type of nAChR mediates the effect of nicotine on tLTP, we made use of three strains of nAChR-knockout mice, each lacking a specific nAChR subunit. Nicotine (10 μM) was applied locally at somato-dendritic regions of the recorded cell from onset to offset of the pairing period (local application protocol II, Methods section), which resulted in a post-synaptic depolarization of similar magnitude during pairing as was observed on wash-in of nicotine (Figs 1f and 3a,b; wash: 4.1±1.0 mV, *n*=9, versus local application: 5.1±1.2 mV, *n*=7). In L6 pyramidal neurons from wild-type (WT) animals, EPSP+AP pairing in the presence of locally applied nicotine resulted in a significantly larger change in EPSP slope (+40±11%, *n*=7) than in control experiments where nicotine was not applied (Fig. 3b,f; −2 ± 8%, *n*=8; one-way analysis of variance (ANOVA): F(1,13)=10.129, *P*=0.007).

In neurons from mice lacking α7-nAChR subunits (α7−*/−*), the depolarization induced by local application of nicotine during pairing was only marginally and not significantly smaller than in WT neurons (Fig. 3b,c; WT: 5.1±1.2 mV; α7*−/−*: 4.9±1.4 mV, *n*=8). In cells lacking α7-nAChRs, the nicotinic facilitation of tLTP was not different from tLTP facilitation in WT control cells (Fig. 3c,f; control: +16±6%, *n*=8; nicotine: +46±11%, *n*=8; one-way ANOVA: F(1,14)=6.418, *P*=0.024). In contrast, in mice lacking β2-nAChR subunits (β2*−/−*), postsynaptic responses to nicotine were completely abolished (Fig. 3d; depolarization 1.2±0.7 mV, *n*=3), confirming that β2-containing nAChRs are indeed the principal nAChRs mediating the postsynaptic depolarizing response to nicotine, as shown previously^11^. In the absence of β2 subunits, the effect of nicotine on tLTP was absent as well (Fig. 3d,f; control: +22±8%, *n*=6; nicotine: +14±10%, *n*=7; one-way ANOVA: F(1,11)=0.316, *P*=0.585), indicating that facilitation of tLTP at L6 synapses relies on β2-containing nAChRs and does not require α7-containing nAChRs.

PFC L6 pyramidal neurons also express α5 nAChR subunits^12^. The α5 subunit confers a higher Ca^2+^ permeability^33^ and reduced desensitization by nicotine to the receptor^12^,^13^,^34^. L6 pyramidal neurons of mice lacking α5-nAChR subunits (α5*−/−*) were still responsive to nicotine via remaining β2-containing nAChRs, but depolarizations were severely reduced compared with WT animals (Fig. 3b,e; WT: 5.1±1.2 mV, *n*=7; α5*−/−*: 2.8±0.6 mV, *n*=11). Nicotine application during EPSP+AP pairing no longer led to facilitation of tLTP in these neurons (Fig. 3e,f; control: +5±12%, *n*=6; nicotine: +15±15%, *n*=11; Mann–Whitney *U*-test, *P*=0.884). Altogether, these results show that β2- and α5-containing nAChRs mediate nicotinic facilitation of tLTP in L6 pyramidal neuron synapses.

### α5-nAChRs are expressed at soma and dendrites of L6 neurons

**Figure 4:**
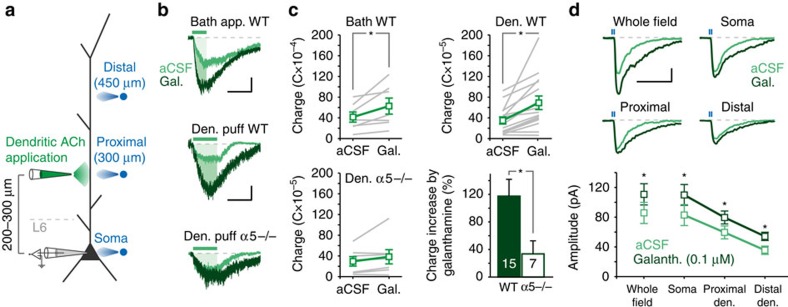
α5-nAChRs are expressed at the soma and in the dendrites of L6 pyramidal neurons. (**a**) Recording configurations. Left: local acetylcholine application (1 mM, 10 s) at the apical dendrite, 200–300 μm from soma (local application protocol III, see Methods) in WT or α5*−/−* animals (**b**). Right: positions of the optic fiber for localized light-induced optogenetic ACh release along apical dendrite of layer 6 pyramidal neurons of *Chat-Cre/Ai32* mice (**d**). (**b**) Responses to ACh bath application (top), and ACh local application at apical dendrites in WT (middle) or α5*−/−* animals (bottom), aCSF conditions (light green) and galanthamine (1 μM, dark green). Top and middle traces from the same neuron. Shaded areas: window for calculating charge transfer (**c**). Top scale bars, 40 pA, 50 s. Bottom scale bars, 20 pA, 10 s. (**c**) ACh-induced charge transfer in aCSF versus galanthamine for ACh bath application (top left panel, *n*=7 (four animals), paired *t*-test, *P*=0.043), and dendritic application in WT (top right panel, *n*=15 (nine animals), paired *t*-test, *P*=0.002) and α5*−/−* animals (bottom left panel, *n*=7 (four animals), paired *t*-test, *P*=0.194). Mean±s.e.m. in green. Bottom right: percentage change in charge transfer by galanthamine in WT and α5*−/−* animals (mean±s.e.m.; independent samples *t*-test, *P*=0.041). (**d**) Augmentation of light-evoked nAChR-mediated currents by galanthamine. Top: current responses to light-evoked ACh release (two pulses, 25 Hz) in aCSF (light green) and with galanthamine (0.1 μM, dark green). Averages of 2–3 trials. Scale bars, 40 pA, 1 s. Bottom: summary showing current amplitude (mean±s.e.m.) for whole field light stimulation (*n*=10 (three animals), paired *t*-test, *P*=0.001), and light from optic fiber at soma (*n*=11 (three animals), paired *t*-test, *P*=0.005), proximal dendrites (*n*=11 (three animals), paired *t*-test, *P*=0.006) or distal dendrites (*n*=11 (three animals), paired *t*-test, *P*=0.002).

Since in the experiments above glutamatergic synaptic inputs were stimulated along the apical dendrite, and since the facilitation of tLTP depended on α5-containing nAChRs, we wondered whether α5-containing nAChRs are actually expressed at apical dendrites. To test this, local applications of ACh (1 mM, 10 s) were delivered either to the soma or to the apical dendrite at 200–300 μm distance from the soma of L6 pyramidal neurons (Fig. 4a; local application protocol III, Methods section). With synaptic transmission blocked (GABAzine (10 μM), DNQX (10 μM)), ACh-induced current responses in aCSF were then compared with those measured in the presence of bath-applied galanthamine (1 μM), which is an allosteric modulator of α5-subunit containing nAChRs that potentiates their currents^12^,^13^,^35^,^36^. In line with these reports, postsynaptic currents evoked by brief bath application of ACh (1 mM, 30 s) were significantly larger in the presence of galanthamine than in aCSF conditions (Fig. 4b (top traces) and Fig. 4c (top left panel); aCSF: 41.5±9.8 × 10^−4^ C versus galanthamine: 62.8±15.4 × 10^−4^ C, *n*=7; *P*=0.044). Galanthamine amplified currents evoked by local ACh applications aimed at the apical dendrite as well (Fig. 4b (middle traces) and Fig. 4c (top right panel); aCSF: 34.9±6.8 × 10^−5^ C versus galanthamine: 69.1±13.1 × 10^−5^ C, *n*=15, *P*=0.002). To verify whether this amplification was truly due to galanthamine acting on α5-subunit containing nAChRs, we performed the same dendritic application experiments in mice lacking the α5 subunit. In these animals, galanthamine did not significantly enhance dendritic currents (Fig. 4b (bottom traces) and Fig. 4c (bottom left panel); aCSF: 29.6±9.1 × 10^−5^ C versus galanthamine: 38.4±13.7 × 10^−5^ C, *n*=7, *P*=0.194), and the potentiation by galanthamine was strongly and significantly reduced (WT: +117.4±24.4% versus α5*−/−*: +33.6±19.0%, *P*=0.041), indicating that galanthamine modulation indeed involves the α5 nAChR subunit. Altogether, these results show that α5-containing nAChRs are expressed along the (apical) dendrites of L6 pyramidal neurons.

To test whether dendritic α5-containing nAChRs are activated by endogenous ACh sources in the mPFC, we used *Chat-Cre/Ai32* mice that express ChR2 in chat-positive neurons^37^ and evoked ACh release with light in control conditions and in the presence of galanthamine (0.1 μM; Fig. 4a,d). Exposing the whole slice to two pulses of blue light (25 Hz) evoked strong inward currents, which were significantly enhanced by galanthamine (Fig. 4d; aCSF: 80.5±14.7 pA versus galanthamine: 110.7±20.1 pA (37.4% increase), *n*=10, *P*=0.001), indicating that cholinergic fibers in mPFC can recruit α5-containing nAChRs. To examine whether cholinergic fibers also targeted α5-containing nAChRs in the dendrites, we next delivered a restricted spot of blue light through an optic fiber placed at a perpendicular angle to the neuron's apical dendrite, for localized activation of endogenous ACh release^29^(Fig. 4a, Methods section). Blue light was then delivered to apical dendrites at distinct locations along the soma-pia axis of L6 pyramidal neurons (Fig. 4a,d). Galanthamine significantly amplified currents evoked by light aimed at soma (aCSF: 82.8±14.0 pA versus galanthamine: 109.9±20.5 pA (32.1% increase), *n*=11, *P*=0.005), proximal dendrites (aCSF: 59.5±8.7 pA versus galanthamine: 79.6±13.7 pA (29.2% increase), *n*=11, *P*=0.006), and distal dendrites (aCSF: 35.5±5.6 pA versus galanthamine: 54.1±9.6 pA (54.8% increase), *n*=11, *P*=0.002). Taken together, both the local, dendritic ACh application and the local light delivery experiments indicate that cholinergic fibers in the mPFC can activate α5-containing nAChRs on apical dendrites of L6 pyramidal neurons.

### nAChR activation enhances dendritic AP propagation in L6

**Figure 5:**
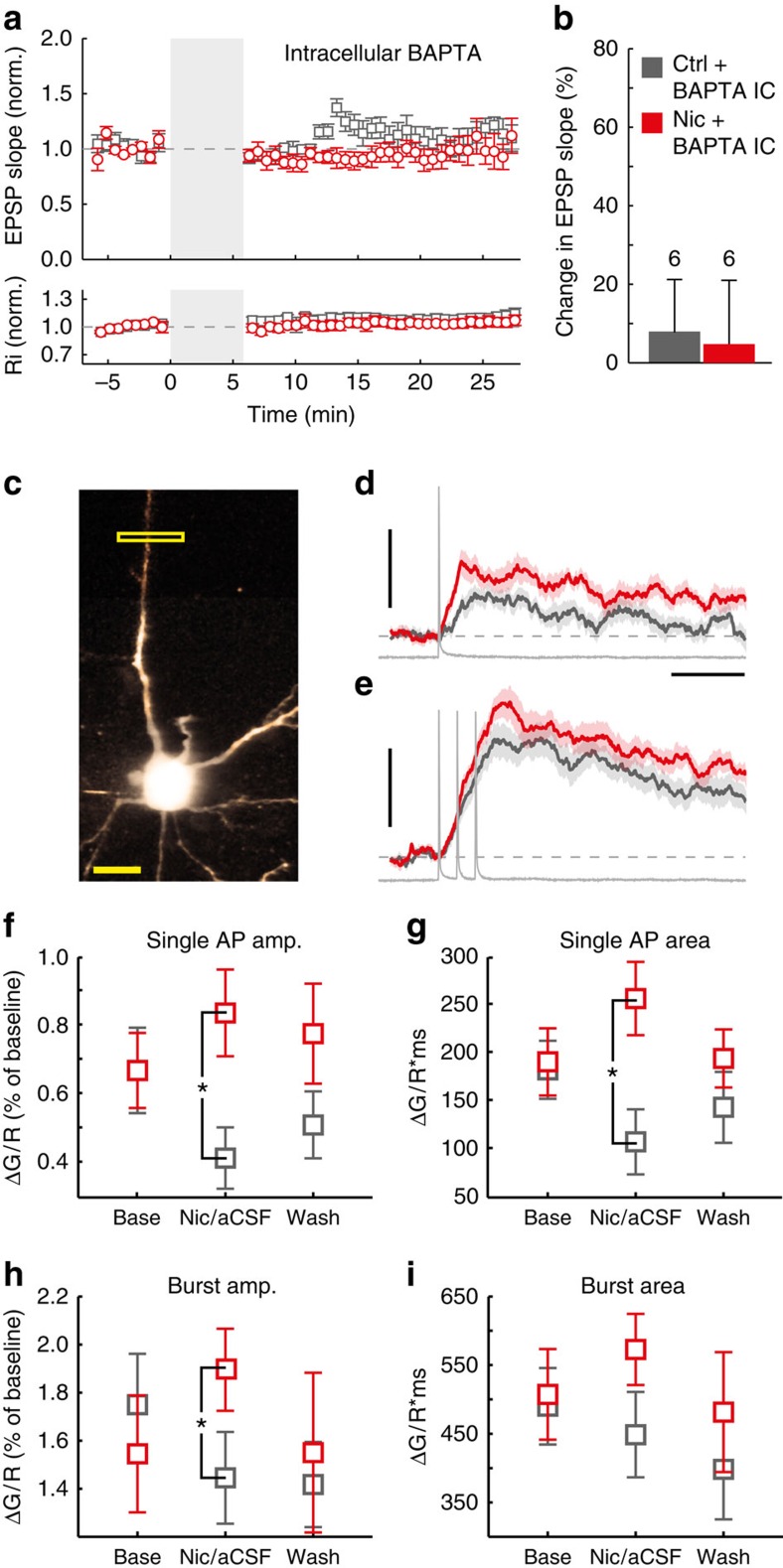
Nicotine amplifies AP-induced dendritic calcium signals in L6 pyramidal neurons. (**a**) Summary of normalized EPSP slope (top panel) and input resistance (bottom panel) data of control (open grey square) and nicotine (open red circle) tLTP experiments where BAPTA (1 mM) was included in the intracellular solution. (**b**) Summary bar chart of tLTP experiments with intracellular BAPTA (mean±s.e.m.). One-way ANOVA: F_(1,10)_=0.023, *P*=0.882; aCSF: *n*=6 (four animals), nicotine: *n*=6 (four animals). (**c**) 2-photon Z-stack of L6 pyramidal neuron with Alexa-594. Boxed in yellow: line-scan location. Scale bar, 20 μm. (**d**) Mean waveform of dendritic Ca^2+^ transients by single AP back-propagation in aCSF (*n*=16 (seven animals); grey trace) or bath-applied nicotine (10 μM, *n*=13 (six animals); red trace). Scale bars, 1% ΔG/R, 100 ms. (**e**) As **d**, for bursts of APs (aCSF: *n*=15 (seven animals); nicotine: *n*=14 (six animals)). Scaling as **d**. (**f**) Ca^2+^ transient amplitude (mean±s.e.m.) following single APs. Univariate ANOVA F_(1,26)_=8.265, *P*=0.008; aCSF: *n*=16 (seven animals), nicotine: *n*=13 (six animals). (**g**) As **f**, for the area of dendritic Ca^2+^ transients. Univariate ANOVA F_(1,26)_=8.183, *P*=0.008. (**h**) As **f**, for bursts of APs. Univariate ANOVA F(1,26)=8.847, *P*=0.006; aCSF: *n*=15 (seven animals), nicotine: *n*=14 (six animals). (**i**) As **h**, for area. Univariate ANOVA F(1,26)=3.425, *P*=0.076.

Induction of tLTP depends on intracellular calcium signalling^23^,^38^. To investigate whether nicotinic facilitation of tLTP in L6 pyramidal neurons depends on postsynaptic calcium signalling, we added the fast calcium chelator 1,2-bis(o-aminophenoxy)ethane-N,N,N′,N′-tetraacetic acid (BAPTA) (1 mM) to the intracellular medium of the recording electrode and tested its effect on L6 tLTP. In the presence of intracellular BAPTA, no tLTP was induced either in control conditions (+8±13%, *n*=6) or in the presence of nicotine during EPSP+AP pairing (Fig. 5a,b; 5±16%, *n*=6; F(1,10)=0.023, *P*=0.882). These findings show that in L6 pyramidal neurons, tLTP and its facilitation by postsynaptic nAChRs depend on postsynaptic calcium signalling.

When α5-containing nAChRs are activated, they depolarize the cell membrane potential (Figs 1 and 3). Since α5-containing nAChRs are expressed along the apical dendrite and they enhance tLTP of glutamatergic synapses, activation of α5-containing nAChRs may facilitate AP propagation along dendrites and increase dendritic calcium influx. To test whether nicotine affects dendritic AP propagation, we investigated dendritic calcium signalling using two-photon calcium imaging. L6 pyramidal neurons were loaded with Alexa 594 (80 μM) to visualize neuronal morphology and the calcium indicator Fluo-4 (100 μM) to measure changes in dendritic calcium levels. Sections of primary apical dendrites were line scanned at a distance of 100–150 μm away from the soma towards pia (Fig. 5c) before, during and after nicotine application. To control for possible bleaching of the calcium indicator as a result of repeated line scanning, control experiments were performed, in which aCSF was washed-in instead of nicotine. Baseline dendritic calcium levels did not change significantly more in response to nicotine wash-in than aCSF (Median change nicotine:+0.53%, interquartile range=1.33%; Median change aCSF: 0.21%, interquartile range=2.49%, independent samples Mann–Whitney *U*-test: *U*_(32)_=118, *P*=0.719). However, in presence of nicotine, fluorescence transients following AP back-propagation were increased compared with aCSF, having both a greater amplitude (Fig. 5d,f; nicotine: 0.83±0.11%ΔG/R, *n*=13; aCSF: 0.41±0.10%ΔG/R, *n*=16; *P*=0.008) and larger area (Fig. 5d,g, *P*=0.008). Fluorescence transients following bursts of somatic APs were increased in amplitude (Fig. 5e,h; nicotine: 1.95±0.13%ΔG/R, *n*=15; aCSF: 1.39±0.14%ΔG/R, *n*=14, *P*=0.006), but not in area (Fig. 5e,i; *P*=0.076). These results show that activation of nAChRs in L6 pyramidal neuron dendrites amplify dendritic calcium signals associated with dendritic AP propagation. Since dendritic calcium signalling is required for tLTP induction, enhanced dendritic calcium signals are likely the mechanism underlying the nicotine-induced facilitation of tLTP.

### nAChR distribution in human frontal and temporal cortex

**Figure 6:**
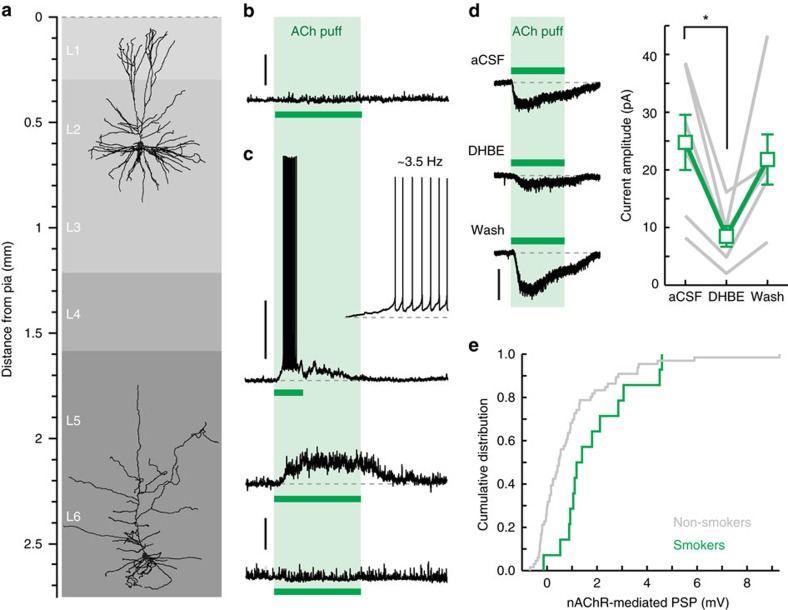
Functional nAChR distribution in human frontal and temporal cortex. (**a**) Example reconstructions of biocytin-labelled human L2/3 and L6 pyramidal neurons (obtained from two different patients). (**b**) Example of the voltage response of human L2/3 pyramidal neuron to local application of ACh (1 mM, 30 s; green bar). Scale bar, 3 mV. (**c**) Examples of voltage responses obtained from different human L6 pyramidal neurons to a local application of ACh. Top inset: magnification of initial segment of AP-firing response. Scale bar, 30 mV (top trace) and 3 mV (middle and bottom trace). (**d**) Pharmacology of human ACh-induced currents. Left traces: current responses to local ACh application recorded from one neuron in aCSF (top trace), in presence of DHβE (middle trace), and after >15 min. wash-out of DHβE (bottom trace). Scale bar, 40 pA. Right panel: amplitude of ACh-induced inward currents in control aCSF, in presence of DHβE, and after wash-out, for individual experiments (grey) and sample mean (green, mean±s.e.m.). Repeated-measures ANOVA: F_(2,10)_=6.675, *P*=0.014; *n*=6 (two patients). (**e**) Cumulative distribution of nAChR-mediated postsynaptic potential (PSP) amplitudes recorded in L6 pyramidal neurons of smoking patients (green; *n*=14 (three patients)) and non-smoking patients (grey; *n*=67 (seventeen patients)). Note the shift to higher response amplitudes in brains of smokers, consistent with increased surface expression of nAChRs.

Do any of the mechanisms of nicotinic modulation of tLTP occur in the neocortex of the human brain? The laminar pattern of nAChR modulation of mouse cortical pyramidal neurons has now been reported in many cortical areas, including prefrontal, motor, entorhinal and visual cortex^11^,^15^,^29^,^39^, showing that nAChRs more strongly excite pyramidal neurons of the deeper layers (L6 mostly) than those of the superficial layers. Autoradiography studies have shown a laminar distribution of nAChRs in human cortex as well^40^, but until now only cortical interneurons of the human frontal and temporal cortex were shown to express functional *α7*-containing and β2-containing nAChRs^41^,^42^. To test whether human cortical pyramidal neurons share a similar nAChR expression profile to rodents, we recorded from L2/3 and L6 pyramidal neurons (Fig. 6a) of human frontal and temporal cortex tissue resected during epilepsy surgery^43^ (see Methods section and Table 1) and tested them for nAChR expression using direct applications of ACh (1 mM, >20 s; local application protocol II, in the presence of atropine) aimed at somato-dendritic regions of the cell (Fig. 6b). In L2/3, none of the recorded pyramidal neurons responded to ACh (0 out of 6 cells; Fig. 6a,b), similar to mouse L2/3 pyramidal neurons^11^. In L6, however, a subset of pyramidal neurons responded to ACh, with responses varying from modest 2–3 mV depolarizations to suprathreshold AP firing (Fig. 6a,c). The corresponding inward currents in response to ACh application were sensitive to the β2-containing nAChR antagonist DHβE (Fig. 6d), similar to mouse cortex^11^. These results suggest a similar laminar expression profile of nAChRs by pyramidal neurons as observed in the mouse brain.

Autoradiography studies have shown that nAChR expression depends on a person's smoking history^44^,^45^,^46^; smoking increases nAChR levels in the brain, and after quitting smoking these return to pre-smoking levels^44^,^46^. It is however not known whether the up-regulation of nAChRs in smokers in fact leads to increased surface expression of nAChRs. To investigate this, we compared the data obtained from patients who smoked with that of non-smokers, pooling ex-smokers in our patient sample (three patients, all ≥2 years of abstinence) with non-smokers. We found that in smokers, the distribution of nAChR-mediated postsynaptic potentials was significantly shifted towards higher amplitudes (non-smokers: *n*=67 (seventeen patients); smokers: *n*=14 (three patients); Kolmogorov-Smirnov test: *P*=0.004, Fig. 6e). This suggests that the increased abundance of nAChRs found in the brains of smokers, particularly in cortical L6, indeed leads to increased surface expression of functional nAChRs by pyramidal neurons in this layer.

### Layer-specific modulation of tLTP in human cortex by nAChRs

**Figure 7:**
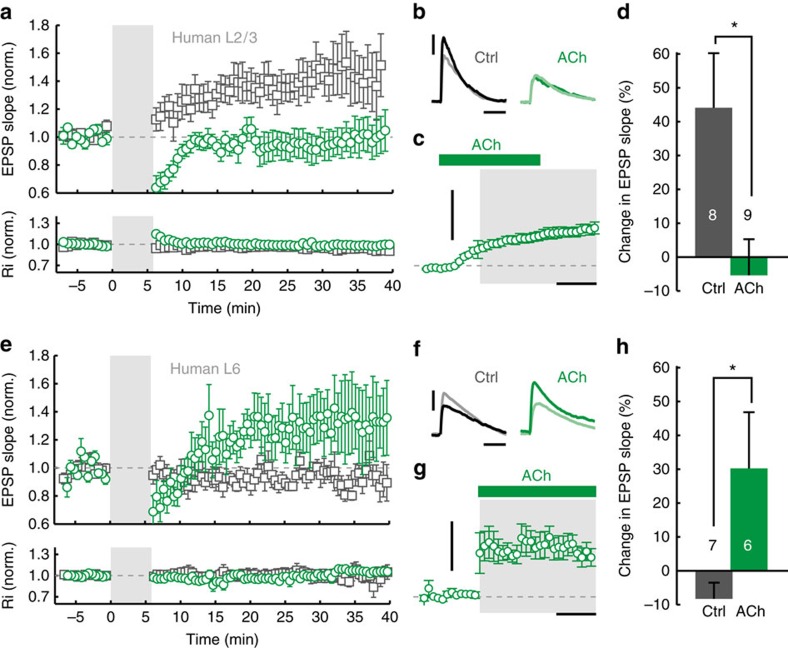
Modulation of synaptic plasticity by nAChRs in human neocortex is layer-specific. (**a**) Summary of tLTP experiments in human L2/3 pyramidal neurons in control conditions (open grey square) and experiments where ACh was present in the bath during pairing (open green circle). Control: *n*=8 (6 patients), Ach: *n*=9 (6 patients). (**b**) Top right traces: Example EPSP waveforms recorded during baseline (light color) and 20–25 min. after pairing (dark color), for tLTP experiments with and without ACh present in bath during pairing. Scale bars, 3 mV, 30 ms. (**c**) Membrane potential change over course of pairing period (grey shading) relative to baseline for experiments where ACh was washed-in during pairing (mean±s.e.m., 14 s bins). Scale bars, 5 mV, 2 min. (**d**) Summary bar chart showing change in EPSP slope of control tLTP and ACh tLTP experiments in human L2/3 neurons (mean±s.e.m.). One-way ANOVA: F_(1,15)_=6.857, *P*=0.019; control: *n*=8 (six patients), Ach: *n*=9 (six patients). (**e**–**h**) As **a**–**d**, for nAChR-bearing human L6 pyramidal neurons. In these experiments, ACh was applied using a continuous local application aimed at somato-dendritic regions of the neuron, from onset to offset of pairing period. One-way ANOVA: F_(1,11)_=5.72, *P*=0.036; control: *n*=7 (seven patients), Ach: *n*=6 (four patients).

To test whether the laminar expression of nAChRs in human neocortex translates into layer-specific nicotinic modulation of synaptic plasticity, similar to mouse PFC, we performed tLTP experiments in L2/3 and L6 of human temporal and frontal cortex. In L2/3 pyramidal neurons, wash-in of ACh (1 mM, in presence of atropine (400 nM)) during pairing resulted in a complete blockade of tLTP compared with control conditions (Fig. 7a,b,d; Δslope ACh: −5±11%, *n*=9; Δslope aCSF: +44±16%, *n*=8; one-way ANOVA, *P*=0.019). These results indicate that tLTP is blocked by nAChR activation in cortical pyramidal neurons of superficial cortical layers, similar to L2/3 and L5 pyramidal neurons in mice^23^,^24^. In mouse L5, nicotine increased the threshold for tLTP by activation of presynaptic interneurons and correspondingly, L5 neurons displayed a mild hyperpolarization of the resting membrane potential following nicotine application^23^. In contrast to mouse L5 neurons, human L2/3 pyramidal neurons slowly depolarized with bath-application of ACh (Fig. 7c; 2.5±0.4 mV, *n*=9), suggesting that distinct mechanisms may be involved in nAChR modulation of tLTP in human L2/3 pyramidal neurons.

Finally, we tested whether tLTP in human L6 pyramidal neurons is subject to modulation by nAChRs by performing plasticity experiments in the subpopulation of nAChR-expressing L6 pyramidal neurons. In control conditions, no tLTP was observed on average (Fig. 7e,f,h; Δslope: −8±5%, *n*=7). Local application of ACh during pairing, which led to a modest but lasting depolarization (Fig. 7g; 2.9±1.7 mV, *n*=6), resulted in an increase of EPSP slope (Δslope: +30.3±16.6%, *n*=6) significantly larger than observed in control conditions (Fig. 7e,f,h; one-way ANOVA, *P*=0.036). Altogether, these results show that the laminar excitation of pyramidal neurons by nAChRs supports layer-specific modulation of human cortical STDP rules.

## Discussion

In this study, we addressed the question whether endogenously released ACh controls plasticity of glutamatergic synapses in a layer-specific manner and what the underlying mechanisms are. We found that (1) in contrast to a suppression of plasticity in layer 5 (ref. 23), postsynaptic β2 and α5 subunit-containing nAChRs expressed by PFC L6 pyramidal neurons facilitate LTP of glutamatergic synapse strength. (2) Endogenous release of ACh can modulate cortical plasticity rules in a layer-dependent manner: tLTP is facilitated in L6 pyramidal neurons, but is suppressed in L2/3 pyramidal neurons. (3) α5 subunit-containing nAChRs are expressed at L6 pyramidal neuron dendrites, are activated by endogenous ACh and increase dendritic calcium influx and AP propagation, which is required for synaptic potentiation in these neurons. (4) In adult human neocortex, nAChRs are also expressed in a layer-dependent fashion in pyramidal neurons. (5) Similar mechanisms that result in layer-specific control of synaptic plasticity by nAChRs in mouse PFC also generate layer-specific modulation of synaptic potentiation in human neocortex. Together, these results show that the innervation of the prefrontal cortex by BF cholinergic neurons and the layer-dependent expression of nAChRs result in a layer-specific control of synaptic plasticity by endogenous ACh. This functional organization of the cortical cholinergic input system is most likely also in place in the adult human neocortex^47^.

Presynaptic nAChRs located on glutamatergic synaptic terminals have been well-known to directly modulate excitatory transmission and plasticity in several brain areas^16^,^17^,^19^,^48^,^49^,^50^. In L5 of the PFC, presynaptic non-α7 nAChRs located on GABAergic interneurons alter synaptic plasticity of glutamatergic synapses on pyramidal neurons by reducing dendritic calcium influx during dendritic AP propagation^23^,^24^. In mouse hippocampus, timing-dependent plasticity can be modulated through a similar recruitment of inhibition by presynaptic nAChRs^51^. nAChR activity could bi-directionally modulate plasticity, and the sign of synaptic change was critically dependent on the timing and localization of nAChR activation. Stimulating α7-subunit-containing nAChRs with a local application of ACh to dendritic regions of the cell during plasticity induction boosts short-term into long-term plasticity^50^,^51^. If however, neighbouring interneurons were activated by nAChRs, the same protocol could no longer induce plasticity^51^. In deep layers of the entorhinal cortex, stimulation of non-α7 nAChRs also boosted short-term to LTP^39^, but neither mechanisms nor nAChR locations were identified. Here, we find that in PFC L6, dendritically located heteromeric nAChRs containing β2 and α5 subunits strongly augment synaptic potentiation of glutamatergic synapses by increasing dendritic calcium influx during dendritic AP propagation. Thus, in different layers of the PFC, dendritic calcium influx in pyramidal neurons is oppositely regulated by endogenous activation of nAChRs either located on the dendrites themselves, in the case of L6 pyramidal neurons, or on presynaptic GABAergic interneurons, in the case of the L5 circuitry. Given that in the mouse and human PFC L2/3 pyramidal neurons typically do not express nAChRs, in contrast to L2/3 interneurons^11^,^41^, activating cholinergic fibers in L2/3 most likely increases interneuron activity and inhibition of L2/3 pyramidal neuron dendrites, thereby inhibiting LTP, similar to L5.

In a study of the dendritic properties of L6 pyramidal neurons of rat somatosensory cortex, it was found that the amplitude of back-propagating APs in apical dendrites of L6 neurons is particularly sensitive to the dendritic resting membrane potential^52^. In light of this, our finding that L6 pyramidal neurons express the tLTP-facilitating nAChRs along their dendrites is interesting, as dendritic nAChRs may well represent a source for such dendritic depolarizations, thereby acting as a physiological on/off switch for bAP enhancement and the induction of tLTP. Note that since the depolarization observed in α5*−/−* animals in response to local application of ACh in dendritic regions is quite similar to that observed in WT animals (Fig. 4c), bAP-induced calcium signalling may be similarly amplified in α5*−/−* animals. In that case, increased calcium influx through the α5-nAChRs expressed by WT animals may act to boost LTP directly.

In the PFC, L6 pyramidal neurons receive fast synaptic cholinergic transmission, but mediated by non-α7 nAChR containing β2 and possibly also α5 subunits^30^. We found here that optogenetic release of ACh at somatic, proximal dendritic and distal dendritic locations activates nAChRs with β2 and α5 subunits that enhance synaptic plasticity. Thus, glutamatergic synaptic plasticity may be augmented by fast cholinergic synapses on L6 pyramidal neurons. Given that distinct BF nuclei may preferentially target either superficial or deep layers of the PFC^7^, L1 and L2/3 interneurons may be innervated by a different population of BF cholinergic neurons than L6 pyramidal neurons. With the layer-specific and neuron-type specific distribution of nAChRs in the PFC^11^ that can take part in fast synaptic cholinergic transmission^30^,^53^, a spatially detailed and millisecond-scale temporal control of PFC glutamatergic and GABAergic signalling and plasticity by the BF cholinergic system is possible.

Although the human neocortex shows structural similarities to the rodent neocortex, many striking differences in cellular and synaptic structure and function have been uncovered in recent years^43^,^54^,^55^,^56^,^57^. Very little is known about whether cholinergic control of cortical processing in the human brain occurs through similar mechanisms as found in the rodent brain. In electron micrographs of the human temporal cortex, 67% of all varicosities on cholinergic axons formed identifiable synaptic specializations on spiny dendrites or spines^58^, which may suggest that fast cholinergic signalling could exist in human neocortex as well. Nicotinic AChRs are abundantly expressed in the human neocortex^40^,^45^,^46^,^59^ and show a laminar distribution with the most dense staining in deep layers^40^,^46^. Cortical interneurons of human frontal and temporal cortex were shown to express functional α7-containing and β2-containing nAChRs^41^,^42^, but whether human pyramidal neurons express functional nAChRs was not known. We found here that similar to mouse PFC, nAChR expression in human pyramidal is layer-dependent. In response to local ACh application, pyramidal neurons in L2/3 did not show nAChR currents, whereas a substantial subset of L6 pyramidal neurons showed prominent inward currents carried by non-α7 nAChRs. Most likely, this layer-specific pattern of nAChR expression underlies the distinct effects of nAChR activation on glutamatergic synaptic plasticity in the human neocortex: suppression in superficial layer pyramidal neurons and augmentation in L6, similar to mouse PFC.

In tLTP experiments in human cortex where ACh was applied during plasticity induction, a reduction in EPSP slope was observed in the first minutes after EPSP+AP pairing (Fig. 7a,e). It is known that in mice, nicotine enhances spontaneous and evoked inhibitory synaptic transmission to L5 pyramidal neurons. The apparent reduction in EPSP amplitude observed during recovery from exposure to ACh may therefore follow from such changes in inhibition/excitation ratio of PSPs evoked by extracellular stimulation. This reduction was most prominent in L2/3 pyramidal neurons; increased inhibition by ACh may therefore underlie the blockade of tLTP in these neurons, as was shown to be the case in mouse L5 pyramidal neurons^23^.

Layer 6 has a prominent role in cortical function. In visual area V1, layer 6 controls the gain of visually evoked activity in neurons of the upper layers^60^. This gain modulation depends on intracortical projections from L6 pyramidal neurons to superficial layers as well as projections to the thalamus^60^,^61^,^62^. PFC L6 pyramidal cells also connect to thalamic nuclei^63^ and play a role in attention and top–down control^64^. How glutamatergic synaptic plasticity in L5 and L6 relates to attention performance and top-down control in mice is not understood at this point. Nevertheless, fast ACh signalling in the PFC at sub-second time scales relevant for nAChR activation during attending and detecting sensory cues^65^ is directly involved in cognitive processing and mediates a shift from monitoring for cues towards the generation of a cue-directed response^66^. It is likely these cognitive processes depend on a balanced laminar control of PFC function by ACh.

## Methods

### Human neocortical brain tissue

All procedures on human tissue were performed with the approval of the Medical Ethical Committee of the VU University Medical Centre and in accordance with Dutch license procedures and the declaration of Helsinki. Human anterior medial temporal cortex and frontal cortex tissue that had to be removed for the surgical treatment of deeper brain structures was obtained with written informed consent of the patients before surgery. Non-pathological neocortical tissue showing no abnormalities on preoperative MRI was obtained from a total of 33 patients (32 adults (17 females, 16 males, aged 19–55 years) and one 9 year-old male), operated for medial temporal lobe epilepsy (15 cases), to remove hippocampal tumours (2 cases), cavernomas (4 cases) or for other reasons (12 cases). Our sample of human patients contained 7 smokers (11.5±3.7 pack years), 23 non-smokers and 3 ex-smokers in abstinence for ≥2 years (Table 1).

### Human and mouse neocortical brain slice preparation

Human brain slices were prepared following the same routines as described previously^43^,^56^,^57^,^67^. Briefly, resected cortical tissue blocks where transported to the laboratory in ice-cold slicing solution containing (in mM): 110 choline chloride, 26 NaHCO_3_, 10 D-glucose, 11.6 sodium ascorbate, 7 MgCl_2_, 3.1 sodium pyruvate, 2.5 KCl, 1.25 NaH_2_PO_4_ and 0.5 CaCl_2_. Transition time between resection of tissue and slice preparation was less than 10 min. Cortical slices (350–400 μm) were prepared in the same ice-cold solution as used for transport, and then transferred to holding chambers containing (in mM): 125 NaCl, 3 KCl, 1.25 NaH_2_PO_4_, 1 MgSO_4_, 2 CaCl_2_, 26 NaHCO_3_ and 10 glucose (solution referred to as artificial cerebrospinal fluid (aCSF) throughout this paper). Here they were stored for ∼30 min at 34 °C and subsequently for at least 1 h at room temperature before recording. All solutions were continuously bubbled with carbogen gas (95% O_2_, 5% CO_2_), and had an osmolarity of ∼300 mOsm.

All animal experimental procedures were approved by the VU University's Animal Experimentation Ethics Committee and were in accordance with institutional and Dutch license procedures. Mouse brain slices were prepared from P19–35 male or female C57BL/6 mice (referred to as WT throughout this paper), from mice lacking either α7-nAChR subunits (α7*−/−*), β2-nAChR subunits (β2*−/−*), or α5-nAChR subunits (α5*−/−*), or from *Chat-ChR(N6)* or *Chat-Cre/Ai32* mice. Following decapitation, the brain was swiftly removed from the skull and placed in ice-cold slicing solution containing (in mM): 125 NaCl, 3 KCl, 1.25 NaH_2_PO_4_, 3 MgSO_4_, 1 CaCl_2_, 26 NaHCO_3_ and 10 glucose. Coronal slices (350 μm) of mPFC were then cut and transferred into holding chambers and allowed to recover in aCSF for at least an hour.

### Electrophysiology in human and mouse neocortical slices

Following recovery, slices were placed in a recording chamber and perfused with aCSF (3–4 ml min^−1^, 31–34 °C). Layer 6 and layer 2/3 pyramidal neurons in human and mouse tissue were identified with oblique illumination or differential interference contrast microscopy. All experiments in mouse tissue were performed in the prelimbic area of mPFC. Whole-cell patch-clamp recordings were then made using standard borosilicate glass pipettes with fire-polished tips (4.0–6.0 MΩ resistance) filled with intracellular solution containing (mM): 110 K-gluconate; 10 KCl; 10 HEPES; 10 K_2_Phosphocreatine; 4 ATP-Mg; 0.4 GTP, biocytin 5 mg ml^−1^ (pH adjusted with KOH to 7.3; 280–290 mOsm). Recordings were made using MultiClamp 700 A/B amplifiers (Axon Instruments, CA, USA), sampling at 10 kHz and low-pass filtering at 3–4 kHz. Recordings were digitized with an Axon Digidata 1440A and acquired using pClamp software (Axon). After experiments were completed, slices were stored in 4% PFA for subsequent neuronal visualization and reconstruction as described in detail in Mohan *et al*^57^.

### Spike timing-dependent plasticity

Spike timing-dependent plasticity experiments were performed using procedures described previously^23^,^43^. Excitatory postsynaptic potentials (EPSPs) were evoked every 7 s (0.14 Hz) using bipolar stimulating electrodes in glass pipettes filled with aCSF positioned ∼100–150 μm along the cell's apical dendrite (Fig. 1a). Duration (50 μs) and amplitude (40–80 μA) of extracellular stimulation were controlled by Isoflex stimulators (A.M.P.I., Jerusalem, Israel). After obtaining a stable baseline of 30–70 EPSPs, spike timing-dependent plasticity was induced within 15 min. of whole-cell by pairing EPSPs to a single postsynaptic AP (50 times, 0.14 Hz, +3 to +8 ms delay), evoked by whole-cell current injection. Timing of EPSPs and APs was controlled by a Master-8 stimulator (A.M.P.I.). The slope of the initial 2 ms of the EPSP was taken as measure of EPSP strength. Change in synaptic strength was defined as percent change in EPSP slope 25–35 min. after onset of pairing relative to baseline. In case recordings lasted less than 20 min. after pairing, the whole post-pairing period (>15 min after pairing to end) was compared with baseline (9 out of 178 included plasticity experiments). Cell input resistance was monitored by applying a hyperpolarising pulse at the end of each sweep (−30 pA in mouse and human L6 neurons, −100 pA in human L2/3 neurons, 500 ms duration). After pairing, membrane potential was returned to approximate baseline value by modest current injection. Criteria for inclusion of recordings in STDP dataset were: (1) baseline resting membrane potential <−60 mV, (2) smooth rise of EPSP and clear separation from stimulation artefact, (3) stable baseline EPSP slope, (4) less than 30% change in input resistance, (5) no AP-firing evoked by extracellular stimulation in post-pairing period. Two cases of extreme EPSP rundown (slope <20% of baseline) were excluded from analysis.

### Light-evoked endogenous ACh release

Endogenous ACh release in *Chat-Cre/Ai32* and *Chat-ChR(N6)* mice was evoked from prefrontal cholinergic fibers with pulses of blue light (470 nm) using a DC4100 4-channel LED-driver (Thorlabs, Newton, NJ). tLTP experiments with light-evoked endogenous ACh release (Fig. 2b–g) were performed in L2/3 and L6 pyramidal neurons of *Chat-ChR2(N6)* mice. Only L6 neurons that depolarized in response to a test pulse of blue light (10 ms duration) were included. The same tLTP protocol as described above was used in these experiments, with the exception that short bursts of light pulses (10 ms duration, 25 Hz) were given before each EPSP+AP pairing (L2/3: five light pulses, starting 200 ms before; L6: 2 pulses, starting 80 ms before). In L6 pyramidal neurons, this caused the EPSP+AP pair to approximately coincide with the peak of the light-evoked nAChR-mediated depolarization. To test whether cholinergic fibers target dendritic nAChRs (Fig. 4d), ChR2 was activated along the dendrites of layer 6 pyramidal neurons of *Chat-Cre/Ai32* mice using an insulated optic fiber (core/cladding Ø 50/125 μm) held within a glass pipette (tip Ø 150 μm). To determine how spatially restricted ChR2 excitation was with optic fiber delivery of light, light-evoked ChR2 currents in ChR2-positive cells were recorded with the light spot at different distances from the cell. These measurements showed that there was a steep drop off of light-induced ChR2 currents when moving the light spot away from the cell. At 400 μm distance from the cell, less than 10% of the ChR2 current remained (data not shown).

### Pharmacology

In all experiments, recording aCSF contained atropine (400 nM; Sigma-Aldrich) to block the actions of muscarinic ACh receptors when ACh was applied, or in case of nicotine application, to prevent muscarinic receptor activation by nicotine-induced endogenous ACh release^68^. This ensured only the actions of nAChRs were measured and provided standardized background experimental conditions for both nicotine and ACh experiments. Other bath-applied drugs were similarly dissolved in aCSF at the desired concentration (nicotine (300 nM in tLTP experiments, 10 μM in two-photon imaging experiments; Sigma-Aldrich), ACh (1 mM; Sigma-Aldrich), Galanthamine (0.1 or 1 μM; Tocris Bioscience), DHβE (10 μM; Tocris Bioscience)). All experiments were performed in the absence of synaptic blockers, except the experiments with light-evoked endogenous ACh release on L2/3 interneurons (Fig. 2a, top traces), the DHβE pharmacology on L6 pyramidal neurons (Fig. 2b), the experiments investigating dendritic expression of nAChRs (Fig. 4) and the DHβE pharmacology experiments on human L6 pyramidal neurons (Fig. 6d), where GABAzine (10 μM; Tocris Bioscience) and DNQX (10 μM; Tocris Bioscience) were included in the aCSF. In the BAPTA-tLTP experiments (Fig. 5a,b), BAPTA (1 mM; Sigma-Aldrich) was added to the intracellular solution.

### Local application of nAChR agonists

Locally applied nAChR agonists were dissolved in aCSF including atropine (400 nM), loaded into glass pipettes and locally applied to neurons by pressure ejection. Three distinct methods of local application were employed in this study. Local application protocol I: ACh (1 mM) was applied for 10 ms using a custom built pulse generator attached to a pressure valve. Local application pipettes had a tip opening of ∼1 μm and were positioned ∼30 μm lateral from soma. Local application protocol II: ACh (1 mM) or nicotine (10 μM) was applied by syringe connected to a local application pipette, continuously at 30–40 mbar pressure for 10–350 s (durations vary per experiment and are specified in main text). Local application pipettes had a tip opening of ∼2 μm and were positioned ∼80 μm lateral from soma. Local application protocol III: ACh (1 mM) was applied for 10 s using a Picospritzer III (General Valve Corporation, Fairfield, NJ). Local application pipettes had a tip opening of ∼1 μm and were positioned ∼30 μm lateral from soma, or 200–300 μm away in the direction of pia, along the apical dendrite. Local application of the fluorescent dye Alexa Fluor 488 showed that the radius of the spread of this type of application in the slice tissue was around 40 to 50 μm. In tLTP experiments on human L6 pyramidal neurons, neurons were categorized as nAChR-positive if the response to ACh using local application protocol I/II was larger than twice the baseline s.d., or -in tLTP+ACh experiments- if neurons depolarized more than 2 mV in the initial phase of plasticity induction in response to local ACh application.

### Two-photon Ca^2+^ imaging

Fluorescent dyes Alexa Fluor 594 (80 μM; Invitrogen) and Fluo-4 (100 μM; Invitrogen) were added to the intracellular solution to visualize morphology and measure [Ca^2+^]_i_ changes respectively within the dendrites. After establishing whole-cell configuration in layer 6 pyramidal neurons, dyes were allowed to diffuse into the dendritic tree for 20 min before imaging. Single APs or bursts of 3 APs (40 Hz) were triggered by somatic current injection (1–2 nA) to induce back-propagating APs (bAPs). bAP-induced Ca^2+^ influx was assessed by the fluorescence change in the Fluo-4 signal relative to the corresponding constant Alexa Fluor 594 signal after background was subtracted from each signal^69^. Fluorescence was measured using a LEICA RS2 two-photon laser scanning microscope with a × 40 (0.8 numerical aperture (NA)) or × 63 (0.9 NA) water-immersion objective and a Ti:Sapphire laser tuned to 830 nm excitation at a bidirectional scanning frequency of 8 kHz. Line scans (500 ms duration, 8 bit signal) synchronized with AP stimulation were made at a dendritic region of interest (ROI) 100–200 μm from soma. To assess the effect of nicotine on dendritic bAPs in layer 6 neurons, nicotine (10 μM) was bath applied and identical stimulus protocols and line scans were repeated. Line scans were repeated 3–6 times per stimulus protocol per ROI and were averaged for analysis. Amplitude (mean %ΔG/R within 50 ms of (last) AP) and area (integral of trace (%ΔG/R*ms) from (first) AP to end of line scan (total window: 420 ms)) of fluorescence signal running average were calculated offline. Fluorescence signals with >0.5%ΔG/R baseline s.d. were excluded from the analysis.

### Analysis and statistics

All raw data was analysed using custom Matlab scripts (R2009a, MathWorks) or Clampfit 10.2. Statistical analysis was performed using IBM SPSS statistics 21. Data were tested for normality using the Shapiro–Wilk test. In case of a significant deviation from normal distribution, non-parametric statistical tests were used. Otherwise, the appropriate parametric statistical test as mentioned in main text was used. In all statistical comparisons, *P*<0.05 was taken as level of significance.

### Data availability

The data that support the findings of this study are available from the corresponding author on request.

## Additional information

**How to cite this article:** Verhoog, M.B. *et al*. Layer-specific cholinergic control of human and mouse cortical synaptic plasticity. *Nat. Commun.* 7:12826 doi: 10.1038/ncomms12826 (2016).

## Figures and Tables

**Table 1:** Details of patient sample from which tissue was used in this study.

**#**	**Gender**	**Age**	**Years epilepsy**	**Seizures/month**	**Cause**	**Neocortical area**	**Active smoker**	**Years of abstinence**	**Pack years**
1	F	33	24.9	60	Cavernoma	Temporal	No	—	—
2	F	53	21.2	8	Other	Temporal	No	—	—
3	M	9	7	0.3	Tumour	Temporal	No	—	—
4	M	44	0.6	NA	Cavernoma	Frontal	No	—	—
5	M	55	11.4	5	MTS	Temporal	No	—	—
6	M	40	38.8	12–20	MTS	Temporal	Yes	—	12
7	F	27	5.9	15	Other	Temporal	Yes	—	0.9
8	F	53	52	4	MTS	Temporal	Yes	—	25
9	M	48	19.8	16	Other	Temporal	No	—	—
10	F	48	1.8	NA	Cavernoma	Temporal	Yes	—	32
11	F	19	6.4	4–90	Other	Temporal	No	—	—
12	F	46	29.7	3	Other	Temporal	No	20	5
13	F	40	16.7	30	MTS	Temporal	No	—	—
14	F	31	23.8	8–180	MTS	Temporal	No	—	—
15	F	48	6.8	45	Other	Temporal	No	—	—
16	F	35	33.8	0.7	Other	Temporal	No	—	—
17	M	34	32.9	0–22	Other	Temporal	No	—	—
18	M	54	9	30	Cavernoma	Temporal	No	—	—
19	M	22	14.5	30	Other	Frontal	No	—	—
20	M	38	10.3	6	MTS	Temporal	No	—	—
21	F	40	6.4	210	MTS	Temporal	No	—	—
22	M	44	39.6	8	MTS	Temporal	No	—	—
23	M	35	12.3	0.9	MTS	Temporal	Yes	—	15
24	F	53	42.4	60–90	MTS	Temporal	No	—	—
25	F	41	32.6	4–9	Other	Temporal	No	—	—
26	M	29	6.6	2–9	Other	Temporal	No	—	—
27	F	20	16.7	4–9	Other	Temporal	Yes	—	0.45
28	M	21	13.3	4–9	Tumour	temporal	no	—	—
29	M	40	19	1–30	MTS	Temporal	Yes	—	2.7
30	F	40	24	0–14	MTS	Temporal	No	8	10
31	M	44	19	1	MTS	Temporal	No	—	—
32	M	43	37	0–14	MTS	Temporal	No	—	—
33	F	33	14	150	MTS	Temporal	No	2	NA

F, Female; M, Male; MTS, Medial Temporal Sclerosis; NA, not available.

‘Pack years' is a standardized quantification of tobacco exposure, defined as the number of cigarette packs smoked per day multiplied by the number of smoking years.

**Figure:**
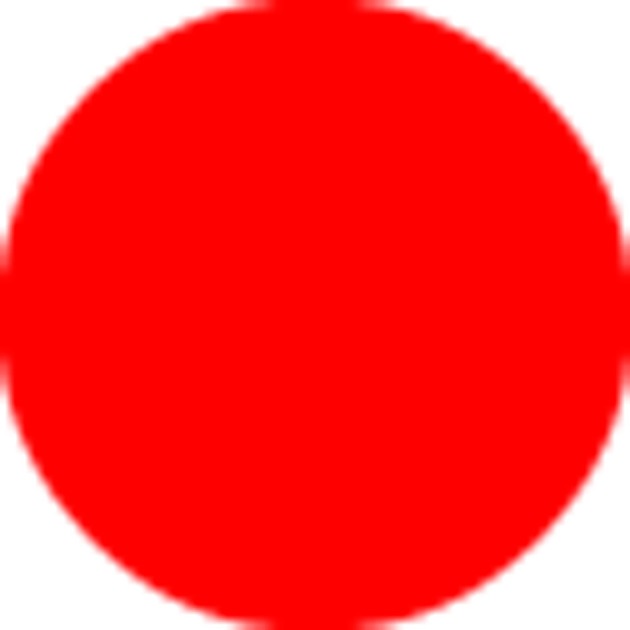


**Figure:**
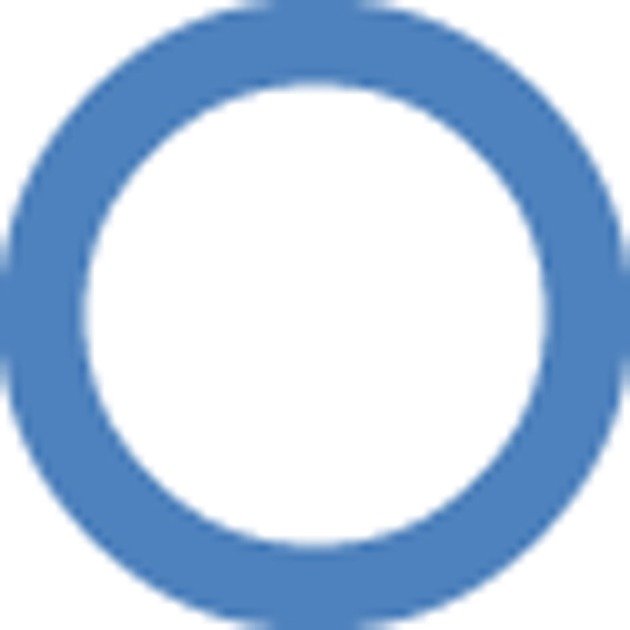


**Figure:**
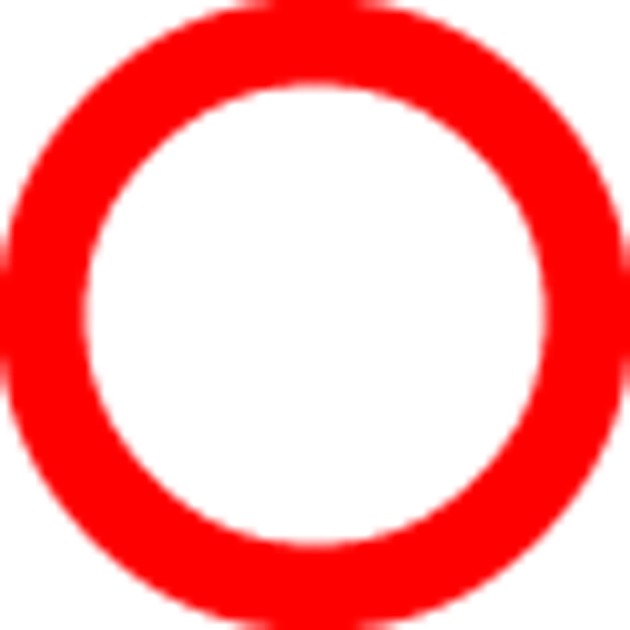


**Figure:**